# Mapping the methylation status of the miR-145 promoter in saphenous vein smooth muscle cells from individuals with type 2 diabetes

**DOI:** 10.1177/1479164116677968

**Published:** 2016-12-21

**Authors:** Kirsten Riches, John Huntriss, Claire Keeble, Ian C Wood, David J O’Regan, Neil A Turner, Karen E Porter

**Affiliations:** 1 Division of Cardiovascular and Diabetes Research, Leeds Institute of Cardiovascular and Metabolic Medicine (LICAMM), University of Leeds, Leeds, UK; 2 Faculty of Life Sciences, University of Bradford, Bradford, UK; 3 Division of Reproduction and Early Development, Leeds Institute of Cardiovascular and Metabolic Medicine (LICAMM), University of Leeds, Leeds, UK; 4 Division of Epidemiology & Biostatistics, Leeds Institute of Cardiovascular and Metabolic Medicine (LICAMM), University of Leeds, Leeds, UK; 5 School of Biomedical Sciences, Faculty of Biological Sciences, University of Leeds, Leeds, UK; 6 Multidisciplinary Cardiovascular Research Centre (MCRC), University of Leeds, Leeds, UK; 7 Department of Cardiac Surgery, The Yorkshire Heart Centre, Leeds General Infirmary, Leeds, UK

**Keywords:** miR-145, DNA methylation, type 2 diabetes, smooth muscle cell, pyrosequencing, saphenous vein

## Abstract

Type 2 diabetes mellitus prevalence is growing globally, and the leading cause of mortality in these patients is cardiovascular disease. Epigenetic mechanisms such as microRNAs (miRs) and DNA methylation may contribute to complications of type 2 diabetes mellitus. We discovered an aberrant type 2 diabetes mellitus–smooth muscle cell phenotype driven by persistent up-regulation of miR-145. This study aimed to determine whether elevated expression was due to changes in methylation at the miR-145 promoter. Smooth muscle cells were cultured from saphenous veins of 22 non-diabetic and 22 type 2 diabetes mellitus donors. DNA was extracted, bisulphite treated and pyrosequencing used to interrogate methylation at 11 CpG sites within the miR-145 promoter. Inter-patient variation was high irrespective of type 2 diabetes mellitus. Differential methylation trends were apparent between non-diabetic and type 2 diabetes mellitus–smooth muscle cells at most sites but were not statistically significant. Methylation at CpGs −112 and −106 was consistently lower than all other sites explored in non-diabetic and type 2 diabetes mellitus–smooth muscle cells. Finally, miR-145 expression per se was not correlated with methylation levels observed at any site. The persistent up-regulation of miR-145 observed in type 2 diabetes mellitus–smooth muscle cells is not related to methylation at the miR-145 promoter. Crucially, miR-145 methylation is highly variable between patients, serving as a cautionary note for future studies of this region in primary human cell types.

## Introduction

miR-145 is a short, non-coding RNA that plays an essential role in differentiation of smooth muscle cells (SMC) from stem cells and maintenance of a quiescent, contractile phenotype in mature SMC.^1^ miR-145 has also been described as a putative tumour suppressor^2^ and a large body of work has highlighted its dysregulation in multiple forms of cancer including prostate, brain and lung.^3–6^ Its potential role in the physiological and pathological development of various malignancies or vascular disorders is therefore undeniable, and a thorough understanding of its regulation would be valuable for informing potential new therapies in many disorders/diseases.

**Figure 1.**
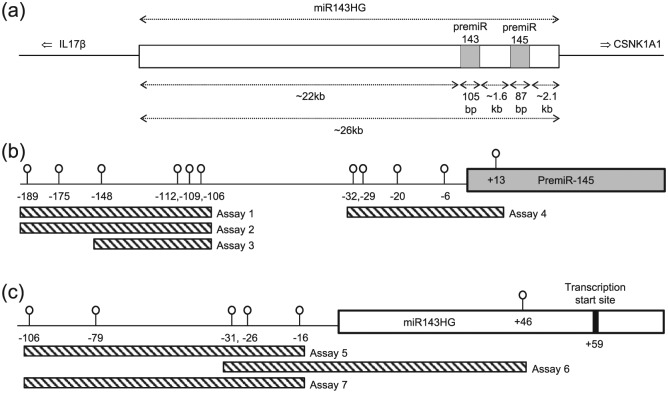
Schematic representation of the miR-145 locus. (a) Both premiR143 and premiR145 (grey boxes) are transcribed from a large primary transcript (miR143HG; white box) from chromosome 5q32, in between the genes encoding interleukin 17 beta and casein kinase 1A1. (b) Eleven potential methylation sites surrounding the premiR145 start site. Specific CpG sites were analysed using multiple pyrosequencing assays, sequences of which can be found in Table 1. (c) Six potential methylation sites flanking the miR143HG coding sequence, with pyrosequencing assays indicated and described in Table 1.

Expression of miR-145 is complex, being encoded in a bicistronic unit with miR-143 on human chromosome 5q32.^7^ A primary transcript (pri-miR-143/145) is generated from the miR-143 host gene (miR143HG, Figure 1) which is then cleaved by DGCR8/Drosha into the two individual pre-miRs, pre-miR-143 and pre-miR-145. These are exported from the nucleus, cleaved by Dicer and incorporated into the RNA-induced silencing complex (RISC) to act on their respective messenger RNA targets (reviewed in Rangrez et al.^7^). A common promoter lies upstream of the miR143HG start site to regulate expression of both miRs;^8^ however, individual promoters for miR-143^9^ and miR-145^2^ have also been described.

**Table 1.** Primer sequences used in pyrosequencing assays.

Region	CpG sites	Primer sequences
miR-145, assay 1	−189, −175, −148, −112, −109, −106	Forward: GAGATTGGGGAATATATATGAGT
		Reverse: [Biotin]-TCTTCTACATCCAACCCCATCTAT
		Sequencing: GAGGGTAGTTTTGGG
miR-145, assay 2	−189, −175, −148, −112, −109, −106	Forward: [Biotin]-GGAGATTGGGGAATATATATGAGT
		Reverse: AAACCAACTAAAATTCTCTTCTACAT
		Sequencing: ATTCTCTTCTACATCCAAC
miR-145, assay 3	−148, −112, −109, −106	Forward: GGTATTTTTTAGGGTAATTGAAGTTT
		Reverse: [Biotin]-TCTACATCCAACCCCATCTATAACAA
		Sequencing: ATTATTTTTTTTTAGAGTAATAAGT
miR-145, assay 4	−32, −29, −20, −6, +13	Forward: GGGTTGGATGTAGAAGAGAATTTTA
		Reverse: [Biotin]-TCCAAAAATCCCCATCTTAACAT
		Sequencing: ATTTTAGTTGGTTTTTAGGGATA
miR143HG, assay 5	−106, 79, −31, −26, −16	Forward: TTTTTTTTTAGGGGGTAAAAGTAATATT
		Reverse: [Biotin]-CCTAACCACAAAAACCACTCTAC
		Sequencing: AGGGGGTAAAAGTAATATTT
miR143HG, assay 6	−31, −26, −16, +46	Forward: GGGGGTGTTTGGGTTAAT
		Reverse: [Biotin]-TAAACCCCCCTCCCCTTAAT
		Sequencing: TTTGGGAGGGTTTAG
miR143HG, assay 7	−106, 79, −31, −26, −16	Forward: GGTTTTTGGTGGTTGGAGTTAAG
		Reverse: [Biotin]-ACCACAAAAACCACTCTACC
		Sequencing: AGGGGGTAAAAGTAATATT

Targeting of messenger RNAs by miRs is a form of epigenetic regulation, whereby gene expression is modulated independently of changes to the DNA sequence. Another form of this regulation, DNA methylation, is also important in directing messenger RNA expression. Due to its involvement in cancer, a number of studies have examined methylation immediately upstream of miR-145.^3–6^ This region contains 11 potential methylation sites (Figure 1(b)), yet while methylation across this region was generally increased in malignant pleural mesothelioma tissue^3^ and prostate cancer sections,^6^ in glioma the scenario was more complex with increased methylation in cell lines^5^ but not in intact tissue samples.^4^ It is therefore crucial to carefully consider source material (i.e. tissues, cell types) when investigating any potential differences in methylation and exploring its relevance to disease pathology.

Type 2 diabetes mellitus (T2DM) is an escalating global burden and an area of intensive research; recent studies have highlighted the importance of differential methylation in this multi-faceted disease.^10–12^ The leading cause of mortality in T2DM is coronary heart disease^13^ and patients frequently require surgical intervention through coronary artery bypass grafting using the patient’s own saphenous vein (SV). However, outcomes in T2DM patients are inferior to those without a diagnosis of diabetes.^14^ Moreover, achieving tight glycaemic control does not ameliorate cardiovascular complications, at least in the medium term.^15,16^ The relative resistance of macrovascular complications to glucose normalisation is suggestive of metabolic memory – a concept believed to possess a strong epigenetic component inducing long-lasting and potentially irreversible changes in cell function.^17^

In support of this theory, we demonstrated potentially detrimental phenotypic changes in a number of cardiovascular cell types derived from multiple patients with T2DM.^18–22^ Specifically, we discovered an aberrant SMC phenotype from the SV of T2DM patients which was characterised by increased spread cell area, cytoskeletal disarray and reduced proliferation, and driven by elevated expression levels of miR-145.^19^ The ability of SMC to dynamically switch and modulate their phenotype is required for adaptive vessel remodelling and is critical following bypass grafting. This persistently aberrant venous phenotype conceivably contributes to the poor surgical outcomes in this patient group, although it is not necessarily representative of global changes within all SMC in the diabetic patient. Importantly, maintenance of the phenotype throughout serial passaging^19,20^ supports the idea of epigenetic regulation at the level of differential miR-145 expression, which itself may be modulated via DNA methylation. Therefore, the aim of this study was to interrogate potential methylation sites immediately upstream of the miR-145 coding region and to determine whether there were any differences between SMC cultured from patients with or without T2DM.

## Methods

### Reagents

All tissue culture reagents were from ThermoFisher (Life Technologies), Paisley, UK, except foetal calf serum (FCS) which was from LabTech International, Uckfield, UK. DNA extraction, polymerase chain reaction (PCR) and pyrosequencing reagents were from Qiagen, Crawley, UK. RNA extraction kit was from Bio-Rad Laboratories, Hemel Hempstead, UK, and miR reverse transcription and Taqman assays were from ThermoFisher (Life Technologies).

### Cell culture

SMCs were isolated and cultured from SV fragments collected from patients undergoing coronary artery bypass grafting at the Leeds General Infirmary as previously described,^23^ with local ethical committee approval (LREC CA01/040) and informed written patient consent. This study conformed to the principles outlined in the Declaration of Helsinki. All cells irrespective of diabetic status were maintained under identical conditions in medium comprising Dulbecco’s modified eagle medium (DMEM) with 10% FCS, 100 µg/mL penicillin-streptomycin, 2 mM l-glutamine and 25 mM glucose, at 37°C in a humidified incubator with 5% CO_2_ in air. All experiments were performed on cells between passages 3 and 5, across which miR-145 expression is stable.^19^

### DNA isolation and bisulphite conversion

DNA was extracted from confluent cells using a QIAamp DNA Micro Kit according to manufacturer’s instructions. Two micrograms of DNA was subsequently converted using EpiTect Plus Bisulfite Kit according to manufacturer’s instructions to convert any unmethylated cytosine residues to uracil residues.

### Pyrosequencing

Pyrosequencing assays were designed using PyroMark^®^ Assay Design SW 2.0 for the region flanking the miR-145 and miR143HG transcription start sites. These included an amplification primer set where one primer was biotinylated, and a sequencing primer. Amplicon length was restricted to <200 bp to ensure optimal pyrosequencing. Bisulphite-converted DNA was amplified using Pyromark^®^ PCR Kit on a thermal cycler as follows: 15 min at 95°C, 45 cycles of 30 s at 94°C, 30 s at 56°C and 30 s at 72°C, then 10 min final extension stage at 72°C (primer sequences are listed in Table 1). A small aliquot was electrophoresed on a 2% agarose gel to ensure a single, intense band was observed. The remaining product was subsequently used for pyrosequencing, in triplicate, on the Pyromark^®^ Q24 according to manufacturer’s instructions. The methylation status of each CpG site was determined using PyroMark^®^ Q24 Software. Standard curves were generated using known proportions of unmethylated and fully methylated control DNA.

### RNA extraction and miR-145 expression

RNA was extracted from 7 × 10^5^ cells using the Aurum Total RNA Mini Kit as previously described.^24^ Expression of miR-145 was determined using Taqman MicroRNA Reverse Transcription Kit and Taqman assays specific to human miR-145-5p and snoU6 (housekeeper) in triplicate according to manufacturer’s instructions.

### Statistical analysis

Data are expressed as mean ± standard error of mean (SEM), with *n* representing experiments performed on different donors’ cells in each group. Patient age was tested for normality in GraphPad Prism v6.05 using the Shapiro–Wilk normality test followed by *t*-test. The proportion of patients of each sex was tested by binomial test. As methylation sites were not acting independently of each other, we used a multivariate model to examine differences at separate sites using the formula

Sites(189,175,148,112,109,106,32,29,20,6,13)~Diabetes+Age+Sex

Diabetes, age and sex were classified as independent variables, and all methylation sites (189 etc.) as dependent variables. Correlation was assessed using *R* squared. Significance was considered at *p* < 0.05. Power calculations were performed using GraphPad StatMate 2.0.

## Results

### Patient demographics

Non-diabetic (ND) patients had a mean age of 59.0 ± 2.5 (range, 40–82) years and were 91% male. T2DM patients had a mean age of 63.5 ± 2.5 (range, 35–85) years and were 87% male. The age of patients in both groups was normally distributed and not significantly different from each other (*p* = 0.21). The proportion of male:female patients was analysed using the binomial test as the number of females in each group was too small to be analysed using chi-square test; again, there was no significant difference in the proportion of males:females (*p* = 0.47). Of the patients receiving treatment for T2DM, 4.3% were diet controlled, 78.3% were receiving oral therapies and 17.4% were additionally supplemented with insulin.

### Methylation assay design

**Figure 2.**
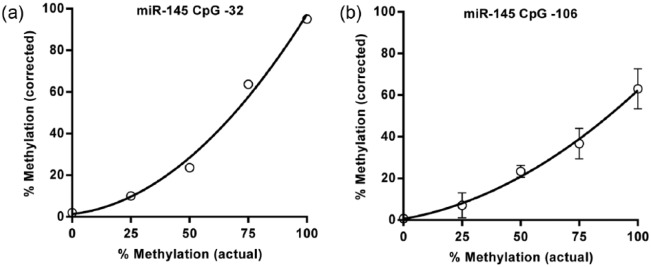
Sensitivity of assays used to interrogate miR-145. Standard curves were generated using methylated controls for all sites, and methylation for all individual SMC populations interpolated from this: (a) standard curve for CpG −32 and (b) CpG −106.

A series of pyrosequencing methylation assays were designed to encompass the miR-145 and miR143HG promoters (Figure 1). The region of interest covering 11 potential CpG methylation sites in the miR-145 promoter was analysed with two primer sets to ensure that pyrosequencing assays were of an optimal length. Using a range of methylated DNA standards (0, 25, 50, 75 and 100% methylated), we characterised the sensitivity of the assays to detect methylation at each position before constructing a standard curve for each CpG site, from which methylation data for individual patients was interpolated. In general, the assays were sensitive to the degree of methylation as exemplified in Figure 2(a) for CpG −32; however, sensitivity at the CpG cluster −112 to −106 was consistently poorer than that at other sites (Figure 2(b)).

### Methylation levels at the miR-145 promoter in SMC isolated from ND and T2DM patients

**Figure 3.**
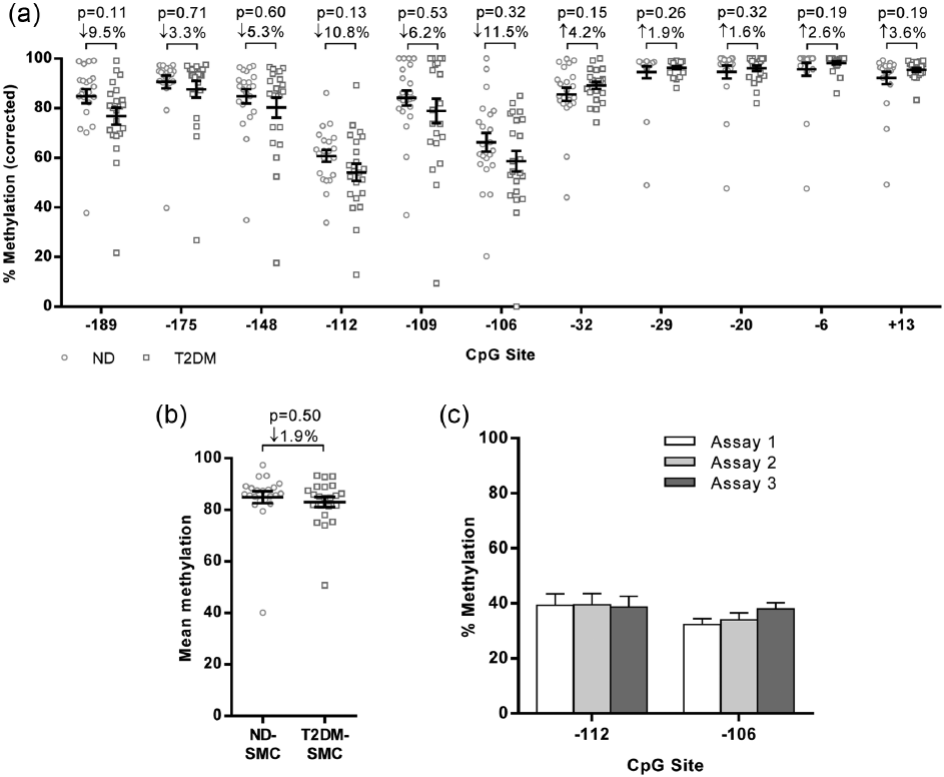
Interrogation of miR-145 methylation in ND and T2DM-SMC. (a) Methylation analysis for all potential methylation sites in both ND and T2DM-SMC (*n* = 22 of each population). (b) Mean methylation across the 11 sites (*n* = 22 of each population). (c) Lower methylation levels at sites −112 and −106 were corroborated using three different assays on DNA from both ND and T2DM-SMC (*n* = 6).

Methylation levels were determined for each site from 22 ND and 22 T2DM-SMC (Figure 3(a)). Overall, there was a tendency to reduced methylation in T2DM-SMC for CpGs distal to the start site (CpG −189 to −106); T2DM-SMC where 2.3%–11.5% lower than ND-SMC. However, at more proximal positions, this pattern was lost and there were no consistent trends in methylation at CpGs −32 to +13 (Figure 3(a)). Inter-patient variation was high at all sites regardless of diabetes; however, the degree of variability was less at CpGs closer to the transcription start site. Given this variation, and despite analysis of a total of 44 patient samples, the study was not sufficiently powered (Table 2) to detect a change in methylation of 5% (the mean difference observed across the 11 separate CpG sites). Analysis of the mean methylation across all 11 sites revealed no significant differences in miR-145 methylation in T2DM-SMC (Figure 3(b)).

**Table 2.** Power calculations to detect a 5% difference between ND and T2DM SMC at individual methylation sites.

Raw data				Required
CpG	Mean	SD	*n*	*n*
−189	84.844	13.458	22	150
−175	90.640	12.041	22	100
−148	84.784	13.614	22	150
−112	60.741	10.992	22	200
−109	84.134	14.493	22	200
−106	66.253	17.628	22	400
−32	85.580	12.795	22	150
−29	94.505	11.350	22	90
−20	94.614	11.976	22	100
−6	95.731	12.144	22	100
+13	92.193	11.465	22	100

SD: standard deviation; ND-SMC: non-diabetic smooth muscle cell.

Power calculations were performed using the standard deviation of ND-SMC at each CpG site. The approximate number of replicates required to determine a difference in methylation of 5% with 80% confidence was calculated.

Methylation at CpGs −112 and −106 routinely appeared lower than at other sites. To ensure these data were robust, we analysed these two positions using three different assays (Figure 1(b) and Table 1) and reliably detected consistently lower levels of methylation at these particular sites (Figure 3(c)).

### Correlation of miR-145 expression with methylation

**Figure 4.**
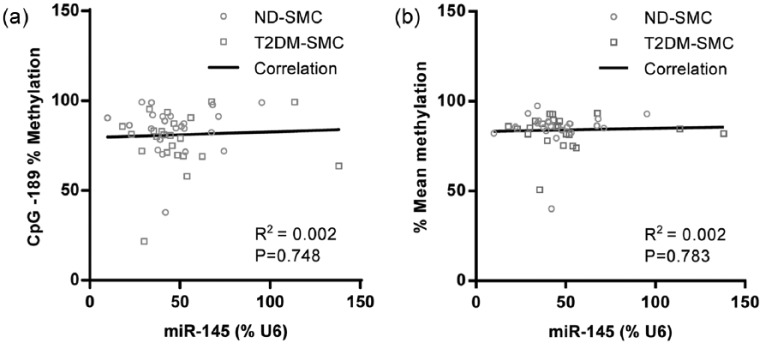
Correlation of miR-145 methylation and expression: (a) correlation of miR-145 expression with methylation at CpG −189 and (b) mean methylation across the entire region (*n* = 22 of each population).

Methylation of this region of the miR-145 gene has previously been reported to correlate with its expression.^6^ Given our previous report of elevated miR-145 expression in T2DM-SMC,^19^ we determined the expression of miR-145 in RNA samples that were prepared in equivalent cells from the same patient cohort. Methylation at any individual site (e.g. CpG −189; the site with the lowest *p*-value of 0.113), and methylation across all sites, did not correlate with miR-145 expression in human SV-SMC (Figure 4(a) and (b)).

### Pyrosequencing of the miR143HG promoter region

As methylation at the miR143HG promoter region could conceivably modulate expression of both miR-143 and miR-145, we proceeded to interrogate six CpG sites (Figure 1, Table 1). However, pyrograms were consistently of a poor quality due to large homopolymeric regions and did not yield reliably quantifiable results (data not shown).

## Discussion

To our knowledge, this is the first comparative study of DNA methylation in primary human SV-SMC in two defined patient cohorts. A key strength was that analysis was performed in cells from multiple patients. Few studies have examined methylation specifically in the SV, and those that have predominantly analysed DNA from whole tissue wherein global changes in methylation in distinct cell types were potentially masked.^25–27^ Using pure populations of human primary cells is therefore a key strength of this study. Other studies have used SMC derived from a single commercial source.^28^ However, this could lead to misinterpretation of any observed differences because, as we have clearly demonstrated here, a single sample is without doubt not representative of a diverse patient population. Our study using isolated SV-SMC from a total of 44 patients provides a broad view of inter-patient variation in methylation that is related to venous SMC specifically.

While the patient donors used in this study were age and sex-matched, we did not have access to full patient demographics [e.g. body mass index (BMI), HbA1c and statins] some of which could conceivably impact our data analysis if they were found to differ between the two groups. However, in our previous, larger study we had access to clinical data on HbA1c, low-density lipoprotein cholesterol (LDL-C) and creatinine for the patient donors and also the proportion who were receiving statin therapy, angiotensin-converting enzyme (ACE) inhibitors, β-blockers, anticoagulant drugs and diuretics. We found that T2DM patients had significantly higher fasting glucose and HbA1c, but all other parameters were identical between ND and T2DM patients including cardiovascular therapies.^19^ Therefore, it is likely that the only known difference between the two patient groups analysed here was a clinical diagnosis of diabetes.

We focussed our attention on the region immediately upstream of miR-145 as this is the only area that has previously been interrogated. We observed trends towards reduced methylation at CpGs −189 to −106. A long-held consensus indicates that promoter methylation and gene expression are inversely related;^29^ the observed subtle reduction in methylation would support this hypothesis, given higher expression levels of miR-145 we reported in T2DM-SMC.^19^ Previous studies demonstrated both hypermethylation and reduced expression in both mesothelioma and prostate cancer.^3,6^ miR-145 modulates cellular proliferation^2,7^ and our observations together with the aforementioned cancer studies support a role for increased miR-145 methylation (and lower expression) in hyper-proliferative disorders such as cancer and reduced methylation (and higher expression) in T2DM-SMC in which we unambiguously demonstrated reduced proliferation.^18–20^

Methylation at CpGs −112 and −106 was consistently lower than at all other sites; this was validated by three separate assays. Reduced methylation at these specific CpGs has only once previously been demonstrated in prostate cancer patients.^6^ Therein, a reduction in methylation at CpGs −112 and −106 was observed in some, but not all, patient samples as we found in this study, suggesting that reduced methylation at these sites might be specific to SMC or non-malignant cells. This highlights the cell-specific nature of DNA methylation^30^ and emphasises the importance of examining methylation in primary, patho/physiologically relevant cell types.

The key finding of this study was the marked variability in miR-145 methylation, irrespective of T2DM. Methylation across the entire region was not statistically different between ND and T2DM-SMC. Retrospectively, using these data we performed power calculations (Table 2) which indicated that a population >200 would be required to achieve statistical power. Because we have previously demonstrated robust, consistent up-regulation of miR-145 expression with sample sizes of 10 per patient population,^19^ the fact that we would require up to 200 for methylation studies adds weight to our argument that differential methylation of the miR-145 promoter is not the causative factor for its elevation in T2DM-SMC.

Since methylation at CpG −189 was most divergent between ND and T2DM cells, we investigated whether this correlated with miR-145 expression. However, methylation at this site alone or across the entire region did not correlate with expression, agreeing with a previous study in glioma.^4^ In contrast, Suh et al.^6^ demonstrated a correlation between methylation and expression in 47 cancer cell lines including breast, colorectal, pancreatic, prostate and kidney; studies that by their nature, minimise inherent variability that is attributable to that in primary cells and tissues.

We previously demonstrated a consistent up-regulation of miR-143 in parallel with miR-145 in T2DM-SMC.^19^ It is plausible that expression and regulation of the host gene miR143HG is more critical for the enrichment of both miR-143 and miR-145, although methylation within the miR143HG promoter has not yet been studied. We attempted a series of pyrosequencing methylation assays upstream of miR143HG; however, the capacity to interrogate this region was overwhelmed by technical issues due to large homopolymeric regions. As a result, pyrosequencing methylation analysis was inconsistent and unreliable. Nevertheless, the ability to develop a robust assay to interrogate the host gene promoter would possibly be valuable.

## Conclusion

In conclusion, pyrosequencing is a robust and accurate technique to quantify DNA methylation at multiple sites in human SV-SMC. A key finding was the very high degree of variation in methylation between cells from different individuals irrespective of diabetes status, underscoring the importance of adequate sample sizes to provide sufficient power when working with material from unrelated individuals and delivering a cautionary note for future studies. In cancer, matched malignant and normal tissue from the same patient almost certainly provides improved statistical power of analysis, and this approach can also be used for atherosclerosis or other localised pathologies;^31,32^ however, it is neither practical nor possible when examining complex, systemic diseases such as T2DM. The high variability that we have shown in methylation makes it incredibly unlikely that changes in methylation are responsible for the persistent and, more importantly, consistent up-regulation of miR-145 expression in T2DM-SMC; however, we cannot discount that methylation of miR143HG may contribute to these differences. Importantly, epigenetic signatures are not limited to DNA methylation – it is likely that a complex interplay between methylation, chromatin and histone modifications and transcription factors are involved in regulating miR-145 expression. While a detailed examination of the miR143HG region may prove informative, studies that adopt a ‘whole population’ approach may not provide the answers being sought. T2DM is a complex heterogeneous pathology that is the culmination of multiple genetic and environmental risk factors. In the future, the advent of personalised medicine whereby a patient is treated on the basis of their unique epigenetic signature may provide the revolution in treatment of T2DM and its complications. Given the increasing epidemic of T2DM and its attendant morbidity, such novel targets for therapy are urgently required.
